# Cytotoxic effects of mineral trioxide aggregate, calcium enrichedmixture cement, Biodentine and octacalcium pohosphate onhuman gingival fibroblasts

**DOI:** 10.15171/joddd.2016.012

**Published:** 2016-06-15

**Authors:** Eshagh A. Saberi, Narges Farhadmollashahi, Faroogh Ghotbi, Hamed Karkeabadi, Roholla Havaei

**Affiliations:** ^1^Assistant Professor, Department of Endodontics, Faculty of Dentistry, Zahedan University of Medical Sciences, Zahedan, Iran; ^2^Assistant Professor, Department of Endodontics, Faculty of Dentistry, Urmia University of Medical Sciences, Urmia, Iran; ^3^Postgraduate Student, Department of Endodontics, Faculty of Dentistry, Zahedan University of Medical Sciences, Zahedan, Iran

**Keywords:** Biodentine, CEM, Cytotoxicity, Human gingival fibroblasts, MTA, OCP

## Abstract

***Background.*** This in vitro study compared the effects of mineral trioxide aggregate (MTA), calcium enriched mixture(CEM) cement, Biodentine (BD) and octacalcium phosphate (OCP) on the viability of human gingival fibroblasts (HGFs).

***Methods.*** After completion of the setting time of the materials under study, fibroblasts were placed in 24-well insert platesand 1 mg of each material was added to the respective wells. The plates were then incubated at 37°C. The inserts were removedat 24, 48 and 168 hours and 2,5-diphenyltetrazolium bromide was added to assess cytotoxicity via the MTT colorimetricassay. Data were analyzed at different time intervals using repeated-measures ANOVA, followed by the Bonferronitest at three levels of significance of P < 0.05, P < 0.01 and P < 0.001.

***Results.*** Cytotoxicity of the materials under study was not significantly different at 24 and 48 hours compared to the controlgroup. However, at 168 hours, a significant difference was noted between MTA (P < 0.05) and Biodentine (P < 0.01)and the control group.

***Conclusion.*** Cytotoxicity of MTA, CEM, Biodentine and OCP against HGFs was similar to that of the control group at 24and 48 hours. Over time, MTA and Biodentine exhibited less cytotoxicity than other materials.

## Introduction

The viability of periradicular cells may be compromised due to the cytotoxicity of materials used during the procedures of pulp capping, repair of perforations and retrograde filling. These materials may induce apoptosis or necrosis of the cells and therefore need to be biologically inert and neutral.^1,2^ MTA yields good biocompatibility results;^3,4^however, its handling and setting time are not ideal.^5^ Recently, new biocompatible materials have been introduced to overcome the shortcomings of MTA. CEM is a biocompatible cement introduced in 2006. It is made up of different calcium compounds. It has easy handling and is capable of forming hydroxyapatite (HA) using an internal source of ions.^6^

CEM has the same clinical applications as MTA. Also, both have the same level of pH, working time and dimensional stability.^6^ However, CEM has a shorter setting time and easier handling.^7^ Also, CEM has greater antibacterial activity than MTA.^8^ CEM enhances the process of stem cell differentiation and induces the formation of hard tissues.^9^ A scanning electron microscope study showed favorable biological response of HGFs to both MTA and CEM.^10^

Histological analyses have shown that the inflammatory responses and biological reactions to CEM and MTA are the same.^11^ However, in contrast to MTA, CEM does not induce cell necrosis after one week.^11^

Biodentine is another calcium silicate cement with dentin-like characteristics, which has been suggested as an alternative to MTA. The manufacturer first introduced BD as an alternative to dentin, inducing the formation of tertiary dentin. The powder and liquid are supplied in one capsule and are mixed in an amalgamator for 30 seconds. The mixture sets after 10 minutes.^12^ The amount of calcium ions released from BD at all time points is higher than that released from MTA.^13^ Only a limited number of studies are available on the biocompatibility of BD. Cytotoxicity testing of BD against HGFs and also 3T3 fibroblasts has yielded results similar to those of MTA.^14,15^ Another material suggested as the direct precursor of HA is the OCP. It has higher potential for stimulation and induction of hard tissue formation than other calcium phosphate cements. It is absorbed over time and replaced with the newly formed hard tissue.^16^

The biocompatibility of synthetic OCP and calcium phosphate ceramic was recently compared in a study, which found OCP to be biocompatible as in other components of HA.^17^

Considering the gap of information on the cytotoxicity of biomaterials, particularly OCP, this study sought to evaluate the cytotoxicity of OCP, BD, CEM and MTA against HGFs.

## Methods

This study was approved by the Ethics Committee of Zahedan University of Medical Sciences (IR.ZAUMS.REC 7058).

HGFs were obtained from the Pasteur Institute of Iran in a medium containing 10% fetal bovine serum (FBS), penicillin, amphotericin B and streptomycin. To adapt to the new environment, the cells in the afore-mentioned medium were incubated at 37°C for 48 hours under 95% humidity and 5% CO_2_. To obtain higher number of cells, the cells were cultured again in a culture medium containing 15% FBS (Gibco, Grand Island, NY, USA) and this process was repeated 5 to 8 times to obtain more cells. This cell line was cultured in a culture medium containing 10% bovine serum (Dulbecco’s Modified Eagle’s Medium, DMEM) in a sterile flask (SPL Life Science, Gyeonggi-do, South Korea). The medium was refreshed every 2‒3 days and the cells were passaged after one week. It should be noted that passage four exhibited the best confluency.

For cell treatment, the insert plates (4.0 μm) (SPL Life Science, Gyeonggi-do, South Korea) were used. To assess the effect of the biomaterials under study, 20,000 cells were cultured in each well of a 24-well insert plate (these plates enable indirect contact of the materials with cells to prevent cell lysis). The cells were incubated (Binder, NY, USA) for 24 hours under 95% humidity and 5% CO_2_. The plates were then removed from the incubator and 1 mg of each biomaterial, i.e. ProRoot MTA (Dentsply Tulsa Dental Specialties, Tulsa, OK, USA), Biodentine (Septodent, France), CEM cement (Bionique Dent, Tehran, Iran) and OCP were prepared in the form of discs under sterile laboratory conditions according to the manufacturers’ instructions and placed in the wells. The plates were retrieved from the incubator at 24, 48 and 168 hours and 10 mL of MTT solution (Sigma Aldrich, St. Louis, MO, USA) and 90 mL of the DMEM culture medium containing 10% FBS were added to plates and incubated at 37°C for 4 hours under 95% humidity and 5% CO_2_. The superficial culture medium in each well was gently extracted and 100 mL of dimethyl sulfoxide (DMSO) (Gibco BRL, Grand Island, NY, USA) was added. After dissolution of formazan crystals, the optical density (OD) of the solution was determined (BioTek, VT, US) at a wavelength of 540‒690 nm. Data were analyzed with GraphPad Prism software (FraphPad Software, San Diego, CA, USA) using repeated-measures ANOVA at different time intervals, followed by the Bonferroni test. The cut-off point for statistical significance was set at P < 0.05, P < 0.01 and P < 0.001.

## 

**Figure 1:**
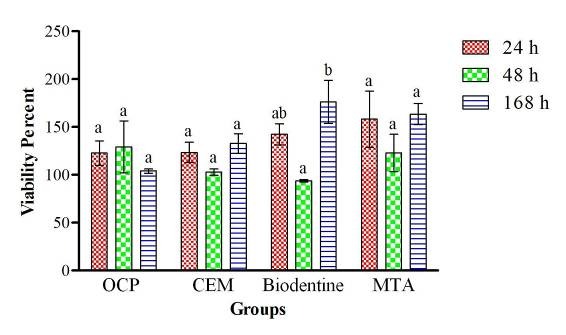
Cytotoxic effects of the biomaterial extracts on human gingival fibroblasts at 24, 48 and 168 hours. 100% cell viability was considered for the control group (medium-only group).

**Figure 2:**
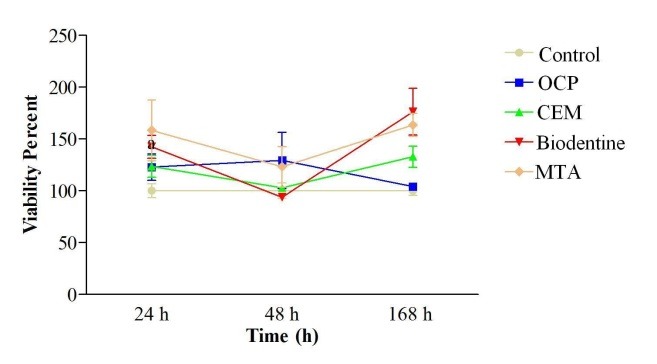
Cytotoxic effects of the biomaterial extracts on human gingival fibroblasts 24, 48 and 168 hours. 100% cell viability was considered for the control group (medium-only group).

Figures 1 and 2 present the viability of HGFs after culture and exposure to MTA, Biodentine, OCP and CEM at 24, 48 and 168 hours.

Assessment of cell viability in BD, OCP, CEM and MTA groups at 24 and 48 hours revealed no significant differences from the control group. However, a significant difference was noted in this regard at 168 hours between the MTA and control groups (P < 0.05) and also between the BD and control groups (P < 0.05). Comparison of the 4 groups with one another revealed no significant differences between any of them at any time interval. OCP was found to have significantly lower cell viability compared to BD at 168-hour interval (P < 0.01). The effect of time on cell viability and cytotoxicity of the biomaterials under study was not significant. Only in the BD group, cell viability significantly increased from 48 to 168 hours (P < 0.01).

## Discussion

Cytotoxicity of the tested materials was not significantly different at 24 and 48 hours compared to the control group. However, there were significant differences between MTA (P < 0.05) and Biodentine (P < 0.01) and the control group at 168 hours.

In this study all the four materials exhibited biocompatibility, although MTA and Biodentine proved superior to the control group at 168 hours. MTT assay was used to assess the cytotoxic effects of MTA, CEM, Biodentine and OCP on HGFs. Being highly calibrated and dependable, the MTT test is also very sensitive (capable of identifying as low as 10^3^viable cells/mL).^18^Fibroblasts are the most numerous connective tissue cells with the capacity to synthesize and maintain connective tissue matrix. The synthesis of collagen types III and I is a main function of fibroblasts in the pulp similar to fibroblasts elsewhere in the body.^19^

In this study, HGFs were used because they are diploid, have higher sensitivity than established cell lines (such as L929 murine fibroblasts) and are a suitable model for early detection of cytotoxicity of root canal filling materials.^20,21^ Also, they have easier culture and higher survival rate than pulp fibroblasts.^22^

In the current study, cell viability in the MTA group increased in the first 24 hours and then decreased in the next 48 hours, followed by an increase again until 168 hours. However, these changes were significant only at 168 hours in the control group (P<0.05). In a study by Ma et al,^23^ gingival fibroblasts exposed to MTA exhibited the highest cell viability at 3 days. At 7 days, a slight reduction in cell viability was noted compared to the control group. Also, in a study by Samara et al^24^on PDL fibroblasts, cell viability increased in presence of MTA during a 0‒72-hour period. Considering the fact that the decrease in pH of MTA over time decreases cell damage and increases cell proliferation,^25^ the significant increase in cell viability in the MTA group at 168 hours in the current study might be attributed to pH variations.

Not many studies have assessed the cytotoxic effects of CEM on HGFs at 168 hours and our study is unique in this respect. Cell viability in the CEM group did not significantly differ from that in the control group at 24, 48 and 168 hours and the highest cell viability was noted in this group at 168 hours.

In a study by Mozayeni et al,^26^ cell viability of L929 fibroblasts exposed to CEM was evaluated at 1, 24 and 168 hours. Similar to our study, they showed higher cell viability in CEM group compared to the control group at all the three intervals; however, this difference was not significant. In contrast to our results, cell viability decreased in their study from 24 to 168 hours but not significantly. Our results found no significant difference in cytotoxicity between CEM and MTA at different time intervals. These results confirm the findings of previous studies indicating the low cytotoxicity of CEM in different cell culture media.^26-29^ The biocompatibility of CEM and MTA depends on their release of calcium ions and the subsequent of bonding of calcium to phosphorous to form HA crystals. These two materials similarly change the enzymatic activity of cells as well as their permeability. This process enhances healing.^28,29^ Consistent with our study, Ghasemi et al^10^ showed in 2014 that HGFs exposed to MTA and CEM were not significantly different in cell viability. They demonstrated that both materials induced the formation of bone morphogenetic protein 2 (BMP-2). BMP-2 attaches to serine/tyrosine receptors and triggers a cascade of signals regulating cell functions such as osteogenesis and cementogenesis. Naghavi et al^28^ reported similar results in their study on L929 murine fibroblasts in 2014 and reported no significant difference in cytotoxicity between CEM and MTA at different concentrations of these materials except at a very high concentration (1000 mg/mL, P=0.019) where the cytotoxic effect of CEM was lower than that of MTA. They attributed this finding to increased release of arsenic in the medium containing very high concentrations of MTA.

We found no significant difference between BD and other groups at 24 and 48 hours. At 168 hours, the highest level of cell viability among groups belonged to BD group. Although it was not significantly different from that in the CEM and MTA groups, the differences between OCP and control groups were significant (P < 0.01). Also, cell viability in the BD group significantly increased from 48 to 168 hours (P < 0.01). Such significant increase in cell viability might be attributed to the properties of BD because the manufacturing process of the active biosilicate technology used for production of BD results in pure calcium silicate and eliminates heavy metal impurities, which are toxic.^14^Moreover, zirconium oxide, serving as radiopacifier in the composition of BD, has no toxic effects on human differentiated cells.^16,30^

Considering the fact that calcium hydroxide peaks in set BD are seen after one day^13^ and also calcium hydroxide has cytotoxic effects in vitro,^31^ the initial drop in cell viability due to exposure to BD may be related to the immediate and abundant release of calcium hydroxide. Similar to our results, Zhou et al^32^ in their study on HGFs found BD and MTA to have no significant difference in terms of cytotoxicity at 1, 3 and 7 days. Corral Nuñez et al^16^ reported similar findings in a study on 3T3 fibroblasts.

However, different results were reported in a recent study on cytotoxicity of tri-calcium silicate-based endodontic materials. In the afore-mentioned study conducted on periodontal ligament fibroblasts, cell viability in the BD group at 3 days was significantly lower than that in the MTA group (P < 0.05); but, at 1 and 7 days, no significant difference was noted between BD and MTA.^33^ Also, cell viability significantly increased from 3 to 7 days, which is consistent with our findings. Zanini et al^34^ evaluated the cytotoxicity of BD against immortalized murine pulp cells and noticed that it initially inhibits cell proliferation in the first 2 days, after which cell proliferation increased similar to the results of our study.

In the current study, no significant difference was noted in viability of fibroblasts in the OCP group compared to that in the control, MTA and CEM groups at different time intervals. These results indicate optimal biocompatibility of OCP. Only at 168 hours, cell viability in the OCP group was significantly lower than that in the BD group. OCP has a chemical formulation of CaOH_2_(PO_4_)5H_2_O and in the current study, OCP was prepared using the method presented by LeGeros.^35^OCP has easy handling and is biodegradable. It has osteoconductive properties and can enhance bone regeneration. Moreover, it is highly soluble.^36,37^

Bodier et al^38^ investigated the effects of calcium phosphate cement on the growth and differentiation of odontoblasts in the dental pulp and found it to be capable of inducing dental pulp cells into odontogenic cells and showed it suitable for pulp capping purposes.

In a study by Sena et al^39^ octacalcium phosphate was used as a pulp capping agent in male Sprague-Dawley rats. They found that reparative dentin formation initiated with a delay in the OCP-based cement group compared to the calcium hydroxide group; however, once begun it developed more rapidly and formed regular dentinal tubules similar to secondary dentin.

High solubility of OCP results in greater effect of its constituents on cells. This can explain lower cell viability in the OCP group at 168 hours compared to the other groups.

Considering the fact that in the current study, complete concentrations of the biomaterials were used for cytotoxicity testing, it appears that evaluating the effects of these materials at different concentrations on other cell types may enhance our knowledge regarding their cytotoxic effects.

## Conclusion

Cytotoxicity of MTA, BD, OCP and CEM was similar to that of the control group against HGFs. However, the cytotoxicity of MTA and BD decreased overtime.

## Acknowledgments

The authors are grateful to the Vice Chancellor for Research of ZAUMS (Zahedan University of Medical Sciences) for the financial support provided.

## Authors’ contributions

EAS and FG were responsible for the main design, concept and interpretation of this article. NF, RH and HK contributed in data acquisition and analysis in the current research. RH drafted the manuscript. All authors critically revised the manuscript. All authors have read and approved the final manuscript.

## Funding

The Funding was provided by Zahedan University of Medical Sciences.

## Competing interests

The authors declare that they have no competing interests with regards to authorship and/or publications of this paper.

## Ethics approval

This study was approved by the Ethics Committee of Zahedan University of Medical Sciences (IR.ZAUMS.REC 7058).
